# Flat panel CT versus multidetector CT in skull base imaging: are there differences in image quality?

**DOI:** 10.1186/s13005-023-00391-0

**Published:** 2023-11-18

**Authors:** Maximilian Schulze, Bernhard Hirt, Katrin Reimann

**Affiliations:** 1 Department of Neuroradiology, University of Tübingen, Hoppe-Seyler-Str. 3, 72076 Tübingen, Germany; 2 Department of Neuroradiology, University Hospital Marburg, Philipps University Marburg, Baldingerstrasse, 35043 Marburg, Germany; 3 Institute of Clinical Anatomy and Cell Analysis, University of Tübingen, Elfriede-Aulhorn-Straße 8, 72076 Tübingen, Germany; 4 Department of Otolaryngology - Head and Neck Surgery, University of Tübingen, Elfriede-Aulhorn-Strasse 5, 72076 Tübingen, Germany; 5 Department of Otolaryngology - Head and Neck Surgery, Philipps-Universität Marburg, Baldingerstrasse, Marburg, 35043 Germany

**Keywords:** Flat panel computed tomography, Multidetector computed tomography, Skull base, Image quality

## Abstract

**Background:**

Purpose of this study was to compare image quality of the skull base in standard 20s protocol flat panel computed tomography (FPCT) with the new time and dose improved 10s protocol as well as with 128 slice multidetector computed tomography (MDCT).

**Methods:**

10 whole skull preparations were scanned with either 128 slice MDCT(SOMATOM Definition AS+, Siemens, Erlangen) or FPCT (AXIOM-Artis, Siemens, Erlangen) using 10s or 20s protocol.

**Results:**

FPCT provides significantly better image quality and improved delimitation of clinically relevant structures in the anterior, temporal and posterior skull base compared to 128 slice MDCT. The 20s FPCT protocol yielded best delimitability of evaluated skull base structures. However, the shorter, dose saving 10s FPCT protocol was still significantly superior to 128 slice MDCT regarding delimitability of skull base structures and additionally showed no significant inferiority compared with the 20s FPCT protocol.

**Conclusions:**

The 10s FPCT protocol yields a significantly better image quality at a comparable radiation dose exposure in imaging skull base structures compared to MDCT.

**Trial registration:**

371/2017BO2.

## Introduction

Minimally invasive surgical access to the skull base has become a widely used technique, as endoscopic equipment and image guided navigation systems are evolving.

While intraoperative navigation systems have been in use since the 1980 for neurosurgical interventions, they are today available in approximately 75% of U.S. hospitals and 25% of European hospitals [15].

Image guided surgery in skull base operations is being used by the majority of neurosurgeons for recurrence operations and extended skull base approach, and from 50% of neurosurgeons in case of complex anatomy, as reported by Esposito et al. in a Global Survey from 2012 [7].

Other conditions where image guided surgery is recommended by The American Academy of Otolaryngology Head and Neck Surgery are changed anatomy due to trauma, pathologies affecting frontal, ethmoidal or sphenoidal sinuses, disease that reaches skull base, orbita, optic nerve or carotid artery [15].

Development in Computed Tomography (CT)-technique has made high spatial resolution imaging together with radiation dose reduction possible by improving image acquisition physics [8], as well as iterative reconstruction software algorithms [18].

Flat Panel CT (FPCT) holds a notably higher spatial resolution than multidetector-CT (MDCT) [12], and is already being used for intraoperative skull base surgery [5] and preoperative imaging.

While image quality of flat panel CT and multidetector-CT in temporal bone imaging has already been compared in various publications, showing an advantage for flat panel CT [3, 22, 24, 25], there are several publications regarding the use of cone beam CT and assessing its accuracy for intraoperative navigation [19, 21]. Comparison between MDCT and Cone beam CT in skull base imaging on phantom and human dry skull showed no significant differences between the 2 CT systems [6]. Another study with focus on the maxillofacial and temporal bone area however showed differences in favor of flat panel CT in comparison with MDCT [2].

The objective of this work is to evaluate image quality of 2 different computed tomography systems, a 128 row multidetector –CT (MDCT) versus a flat panel-CT (FPCT), and the image quality of 2 different FPCT protocols with an acquisition time of 10s and 20s respectively, using 10 whole skull preparations to mimic clinical application.

## Materials and methods

### Whole head temporal bone specimens

10 whole skull preparations were scanned with either 128 slice mdct (SOMATOM Definition AS+, Siemens, Erlangen), or fpct (AXIOM-Artis, Siemens, Erlangen) using 10 or 20s protocol. The study was approved by the institutional research board (IRB) of the Medical Faculty at the Eberhard Karls University Tuebingen. Anatomical specimens were obtained from the body donation program of the Institute of Clinical Anatomy and Cell Analysis of the University of Tuebingen, with written permission of the donors. Additionally at our University every study that is done using anatomical specimens is separately assessed by the local ethics committee.

### CT examinations

Flat Panel CT (FPCT) measurements were performed using a robot-driven C-arm system (AXIOM- Artis, Siemens, Erlangen (Software Version: VC21B 140,128)). Manufacturer-specified programs, 20sDR-H and 10sDR-H, were used to perform a whole head acquisition with following parameters:

20s FPCT: tube potential: 84 kV, tube current: 237mA; dose area product: 6537.4µGym²; frames: 30 F/s; rotation angle: 200°; 98 RAO.

10s FPCT: tube potential: 84 kV, tube current: 246mA; dose area product: 3324.7µGym²; frames: 30 F/s; rotation angle: 200°; 98 RAO.

Slice thickness for both acquisition programs was: 0.139 mm, reconstruction diameter was: 512. A convolution kernel HU (Hounsfield Units) sharp bone was used.

Multidetector CT measurements were performed using a Siemens Somatom Definition AS+. Manufacturer-specified Head protocol was used with following parameters:

Tube voltage: 120 kV, tube current: 220 mAs, collimation: 64 × 0.6 mm, pitch: 0.8 mm, average CT dose index (CTDIvol): 38.93 mGy. DLP: 633 mGycm.

Reconstruction was done at the workstation of the scanner with a high resolution bone kernel: H70h and a FOV of 200 mm, with a slice thickness of 0.6 mm and an increment of 0.6 mm.

### Presence of anatomical structures

Bilaterally present structures were evaluated for each side of the skull base. The craniopharyngeal canal is a midline structure and therefore not bilaterally present.

For inconsistent anatomical structures, frequency of occurrence and lateralization was recorded.

### Quantitative image analysis

For quantitative imaging analysis the signal-to-noise-ratio (SNR) and contrast to- noise-ratio (CNR) were assessed for each CT examination. Two regions of interest (ROI) were placed within the clivus and the cerebellum. Mean Hounsfield Units (HU) and standard deviation (SD) values were measured. The SNR was calculated using the mean value of clivus divided by the standard deviation of clivus;

SNR = mean value_(clivus)_ / SD_(clivus)_.

The CNR was calculated using the difference of mean values of clivus and cerebellum divided by the standard deviation of cerebellum;

CNR= (mean value_(clivus)_ – mean value_(cerebellum)_) / SD_(cerebellum)_.

To test, whether the SNR and CNR of each CT examination differed from zero, a paired Student t test was performed using a significance level of 0.05 (two-tailed).

### Qualitative imaging analysis

For the qualitative analysis of the skull base structures, imaging data of FPCT and MDCT was transferred to Syngo.via multimodality reading software (Syngo.via VB10B, Siemens, Erlangen) for multiplanar reconstruction (MPR) in transversal and coronal planes. Transverse MPRs of FPCT and MDCT were angulated to be parallel to the anterior skull base. Coronal MPRs were angulated to be perpendicular to the transversal MPRs. Sagittal MPRs were angulated to be parallel to the midline of the skull base. According to the in-plane resolution of the different imaging data, slice thickness of multiplanar reconstructions was 0.2 mm for FPCT and 0.6 mm for MDCT. Qualitative image evaluation was performed by an experienced neuroradiologist (M.S.) and an experienced neuro-otologist (K.R.), who were blinded to all subject data and the technical information of the images. The delineation of the structures within the temporal bone was assessed using a 4 point visual analogue scale for image quality with 0 = not delimitable; 1 = poor delimitable; 2 = well delimitable; 3 = very good delimitable. The readers were asked to assess the edge definition for the following anatomical structures of the anterior skull base:

the anterior and posterior ethmoidal canals, the lamina cribrosa, the pterygoid canal, the palatovaginal canal and the inconsistent meningoorbital foramen.

Within the central skull base, readers assessed the delimitability of the foramen rotundum, foramen ovale, foramen spinosum and the inconsistent foramen venosum, as well as the inconsistent midline structure, the craniopharyngeal canal. Additionally, the delimitability of the sulcus of major petrosal nerve was evaluated.

Edge definition in the posterior skull base was evaluated for the following anatomical structures: hypoglossal canal, jugular foramen, the inferior tympanic canaliculus, and the mastoid canaliculus, both inconsistent, as well as the petromastoideal canal and the nerve canal of the facial nerve within the temporal bone respectively.

Anatomical structures were assessed on transversal MPRs, except for lamina cribrosa, foramen rotundum, inferior tympanic canaliculus, mastoid canaliculus, and nerve canal of facial nerve, which were assessed on coronal MPRs, while palatovaginal canal and craniopharyngeal canal were evaluated on sagittal MPRs. All these structures were rated in respect to their delimitation of anatomical boundaries.

### Statistical analysis

Quantitative and qualitative image analysis results are expressed as mean and standard deviation. Qualitative image analysis was evaluated using the Dunn All Pairs Test for Joint Ranks after Bonferroni adjustment for multiple testing, a p-value < 0.05 is considered significant.

Interrater agreement was calculated using the weighted Cohen`s kappa coefficient, classified as: 0-0.20, slight agreement; 0.21–0.40, fair agreement; 0.41–0.60, moderate agreement; 0.61–0.80, substantial agreement; 0.81-1.00, almost perfect agreement [7]. Statistical computations were performed using JMP 14.0.0, © SAS Institute Inc., Böblingen, Germany.

## Results

### Presence of anatomical structures

The evaluated anatomical structures are shown in Table 1.

**Table 1:** Detectability of skull base anatomy. Mean values, standard deviation. Statistical results of Dunn All Pairs Test for Joint Ranks (p < 0.05)

	FPCT 20s	FPCT 10s	MDCT S128	FPCT 20s / FPCT 10s	FPCT 20s / MDCT	FPCT 10s/ MDCT
**Anterior ethmoidal canals**	2.95 (0.15)	2.9 (0.20)	2.4 (0.46)	1.0	< 0.0001*	0.0003*
**Posterior ethmoidal canals**	2.9 (0.30)	2.7 (0.44)	2.0 (0.67)	0.9	< 0.0001*	0.0008*
**Lamina cribrosa**	3.0 (0.0)	2.5 (0.49)	2.0 (0.11)	0.0039*	< 0.0001*	0.0070*
**Pterygoid canal**	2.98 (0.11)	2.85 (0.33)	2.35 (0.56)	1.0	0.0001*	0.0055*
**Palatovaginal canal**	2.78 (0.41)	2.08 (0.52)	1.33 (0.65)	0.0051*	< 0.0001*	0.0173*
**Meningoorbital foramen (incons.)**	2.75 (0.42)	2.67 (0.52)	2.3 (0.76)	1.0	0.6195	0.8774
**Foramen rotundum**	3.0 (0.0)	3.0 (0.0)	2.55 (0.48)	1.0	< 0.001*	< 0.001*
**Foramen ovale**	3.0 (0.0)	2.88 (0.32)	2.5 (0.49)	0.8640	0.0001*	0.0082*
**Foramen venosum (vesalii) (incons.)**	2.96 (0.14)	2.42 (0.49)	1.58 (0.49)	0.0750	< 0.001*	0.0131*
**Foramen spinosum**	3.0 (0.0)	2.52 (0.50)	2.0 (0.49)	0.0126*	< 0.0001*	0.0159*
**Sulcus of major petrosal nerve**	2.93 (0.24)	2.5 (0.49)	1.83 (0.67)	0.0424*	< 0.0001*	0.0092*
**Craniopharyngeal canal (incons.)**	2.0 (0.0)	2.0 (0.0)	1.0 (0.0)	1.0	1.0	1.0
**Hypoglossal canal**	3.0 (0.0)	3.0 (0.0)	3.0 (0.0)	1.0	1.0	1.0
**Jugular foramen**	3.0 (0.0)	2.86 (0.32)	2.85 (0.33)	0.4308	0.1655	1.0
**Inferior tympanic canaliculus (incons.)**	3.0 (0.0)	2.38 (0.48)	1.5 (0.58)	0.5310	0.0151*	0.5310
**Mastoid canaliculus (incons.)**	3.0 (0.0)	2.0 (0.0)	2.0 (0.0)	1.0	1.0	1.0
**Petromastoideal canal**	2.6 (0.50)	2.25 (0.47)	1.6 (0.50)	0.2053	< 0.0001*	0.0073*
**Nerve canal N7**	2.88 (0.32)	2.78 (0.41)	2.35 (0.49)	1.0	0.0015*	0.0154*

Abbreviations: incons.: inconsistant.* indicates significant result (p < 0.05)

In the anterior skull base anterior ethmoidal canals, posterior ethmoidal canals, lamina cribrosa and pterygoid canal were present bilaterally in all skull preparations.

The inconsistent meningoorbital foramen was found bilaterally in 3 skull preparations.

Within the central skull base bilaterally present structures in all skull preparations were the foramen rotundum, foramen ovale and the foramen spinosum as well as the sulcus of major petrosal nerve.

Foramen venosus was present in 7 preparations, of which 6 showed bilateral localizations and one preparation an unilateral localization on the right side.

The inconsistent midline structure craniopharyngeal canal was only present in one skull preparation.

The inconsistent canaliculus innominatus between foramen ovale and foramen spinosum was not present in the evaluated skull preparations.

In the posterior skull base, hypoglossal canal, jugular foramen, petromastoideal canal, and the nerve canal of the facial nerve, within the temporal, bone were present on both sides in all skull preparations.

Inferior tympanic canaliculus was found in 3 preparations, one bilateral and 2 unilateral, both on the left side.

Mastoid canaliculus was only present in one preparation on the right side.

### Quantitative image evaluation

Signal-to-noise ratio decreased from 0.83 (FPCT 20s), to 0.77 (FPCT 10s) and to 0.48 (MDCT).

Contrast-to-noise ratio also decreased from FPCT 20s images to FPCT 10s images to MDCT images, from 0.69 to 0.65 and to 0.39 accordingly (Table 2).

**Table 2:** Quantitative evaluation of signal-to–noise (SNR) and contrast-to-noise (CNR) ratios for FPCT with acquisition of 20s and 10s as well as MDCT. Mean values (SD).

	FPCT 20s	FPCT 10s	MDCT	p-value FPCT20s/ FPCT 10s	p-value FPCT20s/ MDCT	p-value FPCT10s/ MDCT
**SNR**	0.83 (2.98)	0.77 (3.39)	0.48 (1.71)	0.55	0.002*	0.05
**CNR**	0.69 (4.41)	0.65 (4.50)	0.39 (1.83)	0.88	0.32	0.23

SNR differed statistically significant between FPCT 20s and MDCT. Other SNR and CNR values were not significantly different.

### Qualitative image evaluation

Evaluation of subjective image quality showed an interrater agreement for FPCT 20s of 0.81; 95% confidence interval (CI) = 0.68–0.94, for FPCT 10s of 0.84; 95% CI = 0.78–0.91 and for MDCT of 0.88; 95% CI = 0.84–0.94, indicating an almost perfect interrater agreement.

### Comparison of 20s versus 10s FPCT protocol

**Fig. 1:**
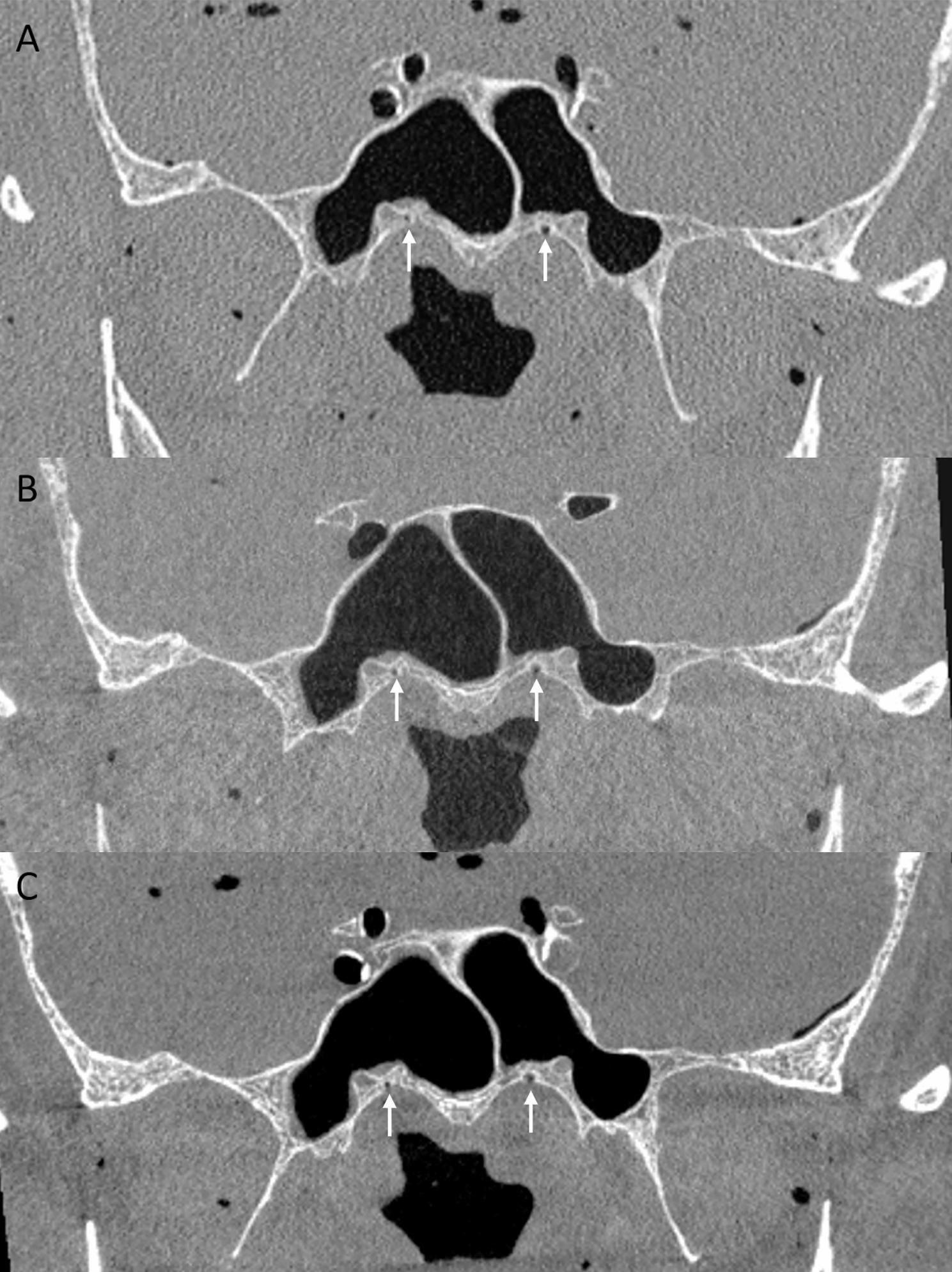
Coronal reformation through anterior skull base with MDCT (**A**), FPCT 10s protocol (**B**) and FPCT 20s protocol (**C**). White arrows indicating palatovaginal canal that courses between the sphenoid process of the palatine bone and the anterior inferior wall of the sphenoid sinus in the roof of the nasopharynx, it transmits the pterygovaginal artery [10]

**Fig. 2:**
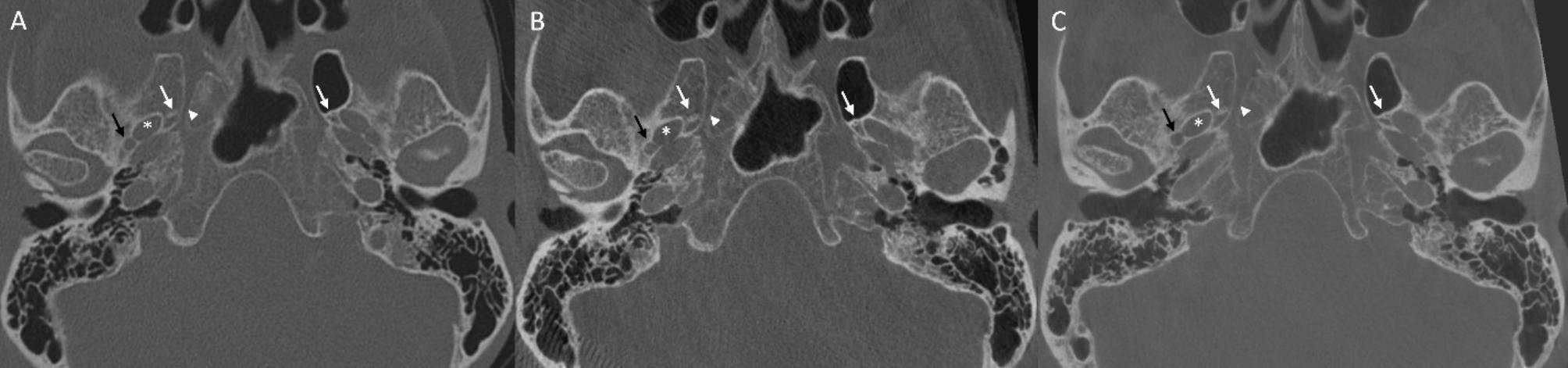
Axial reformation through central skull base with MDCT (**A**), FPCT 10s protocol (**B**) and FPCT 20s protocol (**C**). White arrows indicating foramen venosum; white asterix indicating foramen ovale; white arrow head indicating pterygoid canal; black arrow indicating foramen spinosum

In the anterior skull base, lamina cribrosa and palatovaginal canal (Fig. 1), in the central skull base foramen spinosum (Fig. 2) and the sulcus of major petrosal nerve were significantly better delimitable on 20s FPCT images. All other tested anatomical structures, as well as all structures in the posterior skull base, did not show significant differences between the two FPCT protocols.

### Comparison of 20s FPCT protocol with MDCT

**Fig. 3:**
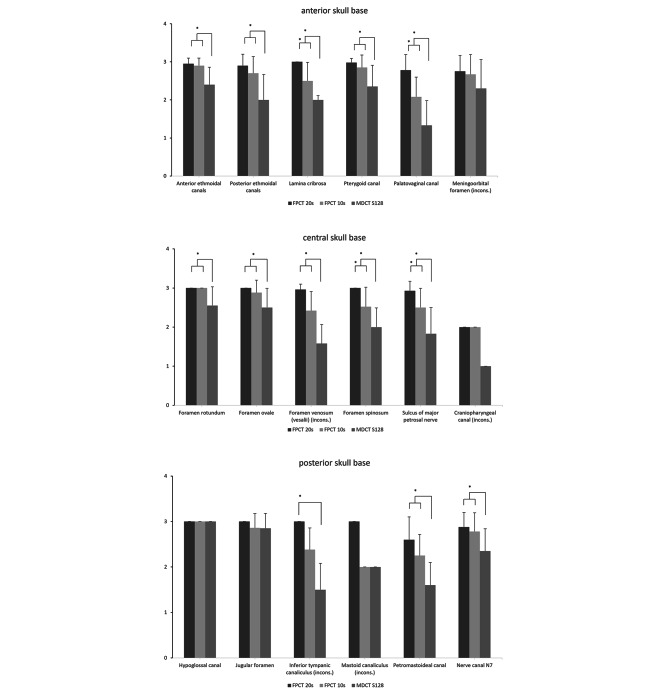
Mean values and standard deviation for detectability with FPCT (FPCT 20s; FPCT 10s) compared to MDCT (MDCT S128) for structures of anterior skull base (**A**), central skull base (**B**), posterior skull base (**C**). *indicates significant difference (p < 0.05, Dunn All Pairs Test for Joint Ranks)

Anatomical structures in the anterior skull base showed significantly better edge definition for all evaluated structures, except for the inconsistent foramen meningoorbitale (Fig. 3A).

In the central skull base, all anatomical structures, except the inconsistent craniopharyngeal canal, which was only present in one whole head preparation, showed significantly superior results for FPCT (Fig. 3B).

**Fig. 4:**
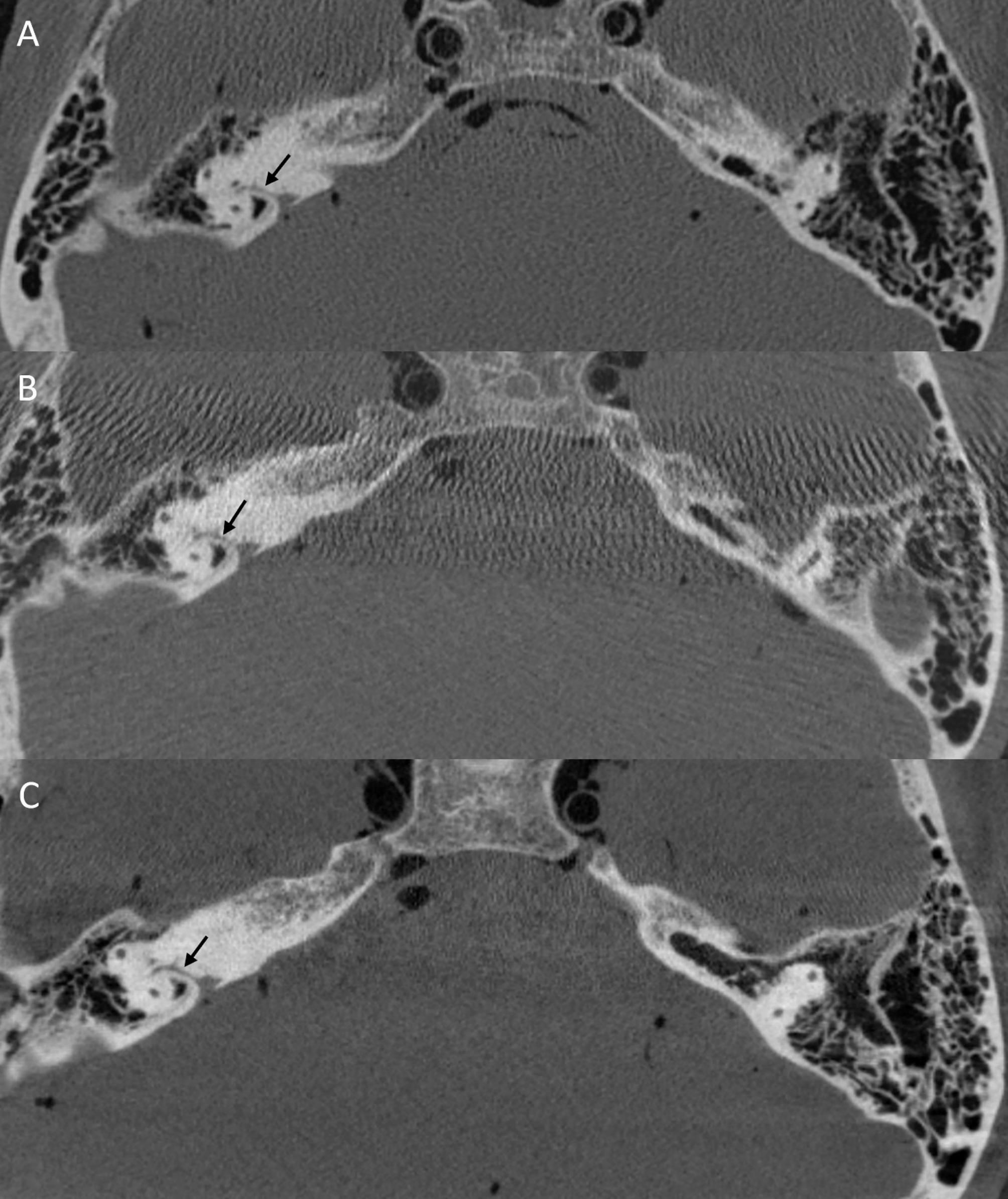
Axial reformation through posterior skull base with MDCT (**A**), FPCT 10s protocol (**B**) and FPCT 20s protocol (**C**). Black arrows indicating petromastoideal canal in the temporal bone

In the posterior skull base, inferior tympanic canaliculus (inconsistent), petromastoideal canal (Fig. 4) and canal of cranial nerve 7 were significantly better delimitable (Fig. 3C).

### Comparison of 10s FPCT protocol with MDCT

Results of significantly better delimitability of anatomical structures correspond to the results of the comparison of 20s FPCT with MDCT, except for the inconsistent inferior tympanic canaliculus, which did not show significant difference. However, delimitability was not as good as for 20s FPCT protocol.

## Discussion

Flat panel CT offers an isotropic spatial resolution which is about two-fold higher than multidetector CT [1].

Imaging of the complex bony and neurovascular anatomy of the skull base profits from high spatial resolution. While some preclinical studies showed no difference in imaging the skull base between MDCT and FPCT [6] or superiority of MDCT over FPCT [2]. Other preclinical studies showed superiority of FPCT compared to MDCT, regarding target registration error for intraoperative navigation in skull base surgery [1], or delimitability of anatomical maxillofacial and anterior skull base structures [14, 20]. Our study showed superiority of two standard FPCT protocols, with different radiation dose, over a standard MDCT-protocol, systematically assessing clinically relevant structures in the skull base of 10 whole head preparations.

The 20s FPCT protocol (20s scan time) yielded best delimitability of bony skull base structures compared with 10s FPCT - which holds half the scan time and half the radiation exposure of the 20sFPCT – and the MDCT protocol. Statistically significant difference in delimitability of nearly all anatomical structures was seen in anterior and central skull base for 20s FPCT and 10s FPCT compared to MDCT, while in posterior skull base differences were not statistically significant between FPCT and MDCT.

Skull base foramina of the middle cranial fossa transmit important neural and vascular structures. The foramen rotundum connects the middle cranial fossa with the pterygopalatine fossa, the second branch of the trigeminal nerve (maxillary nerve) and emissary veins run through it [11].

Asymmetric widening of the foramen rotundum may be a sign of tumoral spread along the maxillary nerve.

The Foramen venosum (Foramen of Vesalius) is an inconsistent communication between the middle cranial fossa and the scaphoid fossa. It transmits a dural sinus, which connects the cavernous sinus with the pterygoid venous plexus [13], its incidence is reported to be 70–80% [11, 16] which is in line with an incidence of 70% in our cohort.

Asymmetry of the Foramen venosum may be due to e.g. carotid cavernosus fistula, tumor and neurofibromatosis [16] or caused by embryological confluence with the foramen ovale.

Foramen ovale forms the communication between the middle cranial fossa and the infratemporal fossa. It carries the third branch of the trigeminal nerve (mandibular nerve), it may contain an accessory meningeal branch of the internal maxillary artery which supplies the trigeminal ganglion (gasserian ganglion) and in the absence of the inconsistent canaliculus innominatus – as in our cohort - it transmits the lesser superficial petrosal nerve, which originates from the tympanic branch of the glossopharyngeal nerve and additionally holds fibers from the facial nerve.

In our cohort foramen ovale was well delimited from neighboring skull base foramina, e.g., foramen lacerum, which sometimes lacks its lateral wall and then communicates with foramen ovale [11].

Foramen spinosum was present bilaterally in all our preparations. It communicates as the foramen ovale between middle cranial fossa and fossa infratemporalis and holds the middle meningeal branch of the external carotid artery, the middle meningeal vein and the recurrent branch of the mandibular nerve [11].

Hypoplasia or absence of the foramen spinosum exists in case of an aberrant middle meningeal artery. This situation arises either due to a fault in embryological development of the stapedial artery, which originates as a branch of the second aortic arch and therefore the internal carotid artery. If the communication between the stapedial artery and the external carotid artery fails to evolve during embryological development, aberrant middle meningeal artery originates from the ophthalmic artery and courses through the superior orbital fissure.

The other cause for an aberrant middle meningeal artery is a persistent stapedial artery which transmits through the tympanic cavity, the facial nerve canal, and the facial hiatus (sulcus of major petrosal nerve) to become the middle meningeal artery.

Clinically important structures in the anterior skull base are the anterior and posterior ethmoidal canals which transmit the anterior ethmoidal artery, vein and nerve and the posterior ethmoidal ethmoidal artery, vein and and nerve respectively. The course of these arteries is variable and therefore important regarding paranasal surgery.

Radiation dose of FPCT has been mentioned by several authors [9, 23].

Struffert et al. showed [26] that FPCT can have a significant dose reduction compared to MDCT standard protocol if collimation is used in FPCT, resulting in the same effective dose of 0.2mSv for 10s FPCT and MDCT, while the effective dose of the 20s FPCT is 0.4mSv.

Therefore, the FPCT with a scan time of 10s can be considered comparable to the MDCT protocol regarding radiation dose in our study.

Reduction of dose results in reduction of signal to noise (SNR) which is in line with the qualitative image rating in our study.

Additionally, contrast to noise (CNR) measurements showed decreased ratios for 10s FPCT and MDCT compared to 20s FPCT, nevertheless all FPCT and MDCT images held diagnostic image quality.

In a recent global survey of usage patterns and the role of intraoperative neuronavigation nearly 25% of skull base surgeons reported using neuronavigation in all cases, main image modalities used were magnetic resonance imaging (MRI) and CT in over 56% of cases [7]. Most indications were complex sinonasal, extended skull base and reoperation cases.

To achieve an accurate navigation, correct registration of CT data to the head of the patient is pivotal. Registration can be done with externally fixed systems (fiducials) or surface registration (e.g., laser or optical) at which externally fixed systems yield the highest accuracy [4, 17]. Taeger et al. showed that a combination of flat-panel volume CT and electromagnetic navigation is highly precise, however there was no significant difference in fiducial registration error (FRE) using a MDCT data set versus a conventional FPCT data set [27].

Nonetheless, FPCT carries in contrast to MDCT the advantage of being available as a mobile device, and therefore being suitable for intraoperative scanning, which allows for adaption of navigation to surgery-related anatomical changes, or robot assisted stereotactic surgery [28].

Comparative studies regarding skull base CT imaging, dealing with image quality and resolution, were predominantly performed in explanted skull base preparations or 3D printing models.

In our study on whole-head preparations, we show that clinically relevant skull base structures are significantly better delimitable using FPCT compared to MDCT.

Regarding the radiation-reduced 10s FPCT protocol, there were only 4 of 18 evaluated anatomical structures significantly inferior delimitable in comparison to the 20s FPCT protocol, while image quality was better than that of MDCT.

In conclusion, 10s FPCT protocol serves as a substantiated tool whenever a high spatial resolution imaging of the skull base is needed.

## Data Availability

The data that support the findings of this study are not openly available due to reasons of sensitivity and are available from the corresponding author upon reasonable request.

## Declarations

The authors state that accepted principles of ethical and professional conduct have been followed.

## Abbreviations

CNR: contrast to- noise-ratio
CT: Computed Tomography
CTDIvol: CT dose index
FOV: Field of view
FPCT: flat panel computed tomography
FRE: fiducial registration error
HU: Hounsfield Units
MDCT: multidetector computed tomography
MPR: multiplanar reconstruction
MRI: magnetic resonance imaging
RAO: Right anterior oblique
ROI: Region of interest
SD: Standard deviation
SNR: signal-to-noise-ratio
U.S.: United States
